# Predictors of discordant latent tuberculosis infection test results amongst South African health care workers

**DOI:** 10.1186/s12879-019-3745-5

**Published:** 2019-02-08

**Authors:** Shahieda Adams, Rodney Ehrlich, Roslynn Baatjies, Nandini Dendukuri, Zhuoyu Wang, Keertan Dheda

**Affiliations:** 1 Division of Occupational Medicine, School of Public Health and Family Medicine, University of Cape Town, Cape Town, South Africa; 2 Department of Environmental and Occupational Studies, Faculty of Applied Sciences, Cape Peninsula University of Technology, Cape Town, South Africa; 3 Division of Clinical Epidemiology, McGill University Health Centre – Research Institute, Montreal, Canada; 4 Centre for Lung Infection and Immunity, Department of Medicine and UCT Lung Institute, University of Cape Town, Groote Schuur Hospital Observatory, H46.41 Old Main Building, Cape Town, 7925 South Africa

**Keywords:** Latent tuberculosis infection, Sensitivity, Specificity, Discordance and health care worker

## Abstract

**Background:**

The tuberculin skin test (TST) and interferon-gamma-release-assays (IGRAs) are utilized in screening programmes for presumed latent tuberculosis infection (LTBI) in health care workers (HCWs). However, inter-test comparison yields high rates of discordance, which is poorly understood. The aim of the study was therefore to identify factors associated with discordance amongst HCWs in a TB and HIV endemic setting.

**Methods:**

505 HCWs were screened for LTBI in South Africa using the TST and two IGRA assays (QuantiFERON-TB-Gold-In-Tube (QFT-GIT) and TSPOT.TB). Factors associated with discordance were analyzed using a multinomial logistic regression model.

**Results:**

TST-IGRA discordance was negatively associated with longer duration of employment for both TSPOT.TB (OR = 0.92; 95% confidence interval (CI) 0.85–0.99) and QFT-GIT (OR = 0.90; 95% CI 0.84–0.96). Marked test discordance occurred in HIV-infected individuals who were more likely to have TSPOT.TB + ve / TST-ve discordance (OR 4.44; 95% CI 1.14–17.27) or TSPOT.TB + ve / QFT-GIT-ve test discordance (OR 5.72; 95% CI 1.95–16.78). Those engaged in home care were less likely to have QFT-GIT + ve/TSPOT.TB -ve / discordance (OR 0.32; 95% CI 0.10–0.95).

**Conclusion:**

The marked TST-IGRA and IGRA-IGRA discordance in HIV-infected individuals suggest greater sensitivity of TSPOT.TB in immunocompromised persons or potential greater reactivity of TSPOT.TB in this population.

## Background

The diagnosis and management of presumed latent TB infection (LTBI) form an important strategy in the fight against TB. The identification of patients with LTBI at high risk of progression to active tuberculosis is deemed a research priority [1]. The World Health Organization (WHO) currently recommends that testing and treatment for LTBI be performed on all HIV positive individuals. This recommendation is extended to populations deemed at increased risk of TB such as health care workers (HCWs) in low TB incidence settings only [2]. However, there are considerable differences in screening guidelines between countries, with limited evidence to support choice and effectiveness of testing strategies [3].

In South Africa, a high tuberculosis (TB) incidence country, the prevalence of LTBI in HCWs as measured by a positive tuberculin skin test (TST) ranges from 48 to 84% while HIV prevalence is > 10% [4]. Despite their high risk for active TB and increased exposure to TB in the workplace, screening for LTBI among HCWs is very limited in practice [5].

Reasons include resource constraints as well as uncertainty about the performance of TST and interferon-gamma release assays (IGRAs). Stigma may also play a role – as it is defined as devaluing a social trait or characteristic which can lead to discrimination of an individual or group. A diagnosis of tuberculosis through its association with poverty and HIV in this setting carries with it a considerable degree of stigma. A recent study of South African HCWs found stigma to be negatively associated with health seeking behavior such as screening for TB screening or presenting for Isoniazid prophylaxis [6].

Despite low quality evidence, IGRAs have been proposed as acceptable alternatives to TST and have been incorporated into screening guidelines by WHO [2, 7–9]. These assays react to early secreted antigenic target 6 (ESAT-6) and culture filtrate protein (CFP-10) encoded within the region of difference (RD1) of the *M tuberculosis* genome. Two IGRAs are available: QuantiFERON-TB Gold-In-Tube (QFT-GIT; format recently changed to QuantiFERON-TB Gold) (Cellestis Limited, Victoria, Australia) and TSPOT.TB (Oxford Immunotec, Oxford, UK). IGRAs have superior specificity and are less affected by cross reactivity with previous BCG vaccination than TST [10, 11]. There is marked discordance in test performance between TST and IGRAs. This phenomenon is partially accounted for by test properties but also appears to be modified by host, immunological and exposure factors. The utility of IGRAs in TB endemic settings has not been shown, and a clearer understanding of factors associated with discordance should result in a more informed approach to screening strategies. To address this knowledge gap we determined the occupational and non-occupational factors associated with TST/IGRA discordance in HCWs.

## Methods

Participants were drawn from five primary healthcare facilities which provide TB diagnostic and treatment services, and two secondary level hospitals caring for patients with complicated, multi-drug-resistant or extremely drug- resistant TB. Five facilities were located in the Cape Town township of Khayelitsha, with a TB case notification rate of over 1600/100000, 70% of which represents with co-infection with HIV [12].

All participants underwent administration of TST and venesection for QFT-GIT and T- SPOT.TB. TST was performed using 1 TU dose of PPD RT23 (Statens Serum Institut, Copenhagen, Denmark). A detailed methods section has previously been published [13]. The value for a positive TST was a skin reaction measured as 10 mm in diameter. This relatively high cut-off was used to improve specificity- as there is near universal vaccination with BCG given at birth in South Africa. For those who were HIV-infected a value of 5 mm was considered a positive TST result to counteract a possible anergic effect due to immunosuppression.

Blood samples for the IGRA assays were drawn concurrently or within 3 days of administering TST. This interval has been shown to be sufficiently short to eliminate potential boosting [14, 15]. The QFT-GIT test was considered positive if the interferon- gamma response minus the nil antigen was ≥0.35 IU/ml. For TSPOT.TB, the number of IFN-λ spot forming T cells (SFC) per million peripheral blood mononuclear cells (PBMCs) was determined using an AIM ELISPOT reader and Oxford Immunotec software. A cut-off of six or more spots was treated as a positive result. To minimize inter-operator and inter-laboratory variability, all assays were done by one operator. Indeterminate results were treated as missing data and observations were not used in the agreement analysis.

Statistical analyses were performed using Stata version 11 (Stata Corp, College Station, Texas). Outcomes included agreement between the tests using the kappa statistic (κ), and factors associated with discordance. Agreement analyses was performed on baseline results and has been reported previously [13].

Discordant pairs numbered six in total: TST + ve/IGRA-ve, TST- ve/IGRA+ve or IGRA+ve/ IGRA-ve. Factors associated with discordance at baseline were explored using a series of multinomial logistic regression models that compared discordant groups with a reference group displaying perfect agreement (negative and positive concordance). Discordance was evaluated between 2 different tests e.g. IGRA and TST or IGRA and IGRA. The association between each variable and two potential discordant test outcomes was then evaluated in one multinomial regression model. A multivariate analysis adjusting for all variables in the model was conducted. Significant covariates in the model were age, gender and HIV status. Variables included in the final model for all three tests were chosen on statistical significance (*p* < 0.05) for any of the three tests in unadjusted analysis, and biological plausibility.

## Results

### Participants with TST and IGRA assay outcomes

**Fig. 1:**
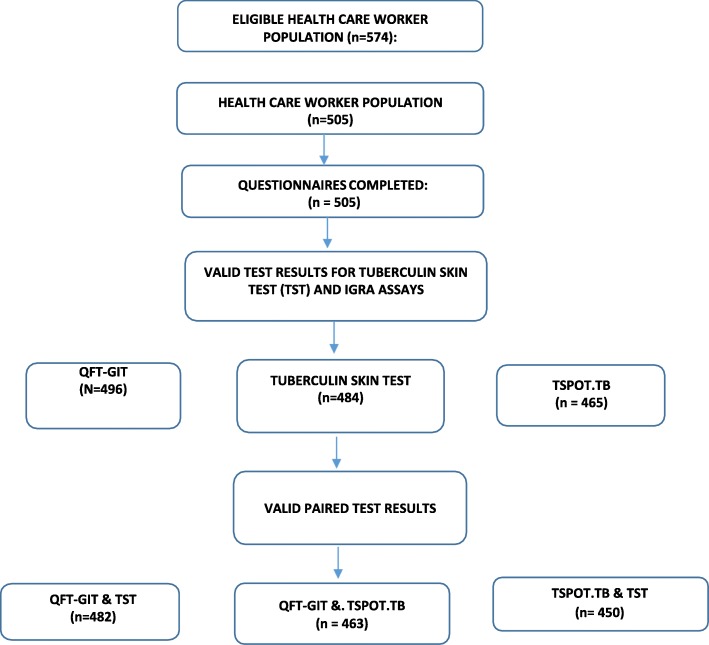
Study plan and flow diagram illustrating valid paired tests for the discordance analysis

The population eligible for participation were 764 HCWs. Based on voluntary participation, 505 HCWs were recruited, a 67% participation rate (Fig. 1). Owing to a higher rate of indeterminate results there were fewer valid test pairs available for comparison for TSPOT.TB (*n* = 450) than for QFT-GIT (*n* = 482).

### Agreement between TST reactions and IGRA assays

Agreement at baseline has previously been reported on for this study [13]. There was only fair agreement between TST and the IGRAs, with κ = 0.28 [95% confidence interval (CI) 0.20–0.36] and κ = 0.25 (95% CI 0.18–0.33) for QFT-GIT and TSPOT.TB respectively.

Agreement between the two IGRA assays was moderate with κ = 0.65 (95% CI). Agreement analysis was performed between the IGRA assays and three different cut-offs for TST (≥5, ≥ 10 and ≥ 15 mm) without taking HIV status into consideration and was essentially unchanged across the different cut points.

### Predictors of discordant test responses

Table 1 presents the results of the multinomial models used to estimate the relative odds of discordant results between QuantiFERON-TB Gold-in-tube and tuberculin skin test. The reference category for all models includes pairs displaying perfect agreement, with both tests giving the same result, either positive or negative.

**Table 1:** Results of multinomial regression models for discordant QuantiFERON-TB Gold-in-tube and tuberculin skin test at baseline

	Positive TST and negative QFT-GIT test result		Negative TST and positive QFT-GIT test result	
Risk Factor	OR (95% CI) *N* = 112	Adjusted	OR (95% CI) *N* = 24	Adjusted
Older age, per each additional year	0.99 (0.70–1.01)	1.00 (0.97–1.02)	1.01 (0.98–1.05)	1.08 (1.03–1.13) **
Male gender	0.93 (0.57–1.52)	0.75 (0.44–1.28)	1.15 (0.46–2.87)	1.07 (0.38–3.04)
BCG Vaccination scar	1.28 (0.74–2.21)	1.27 (0.69–2.31)	1.06 (0.39–2.92)	0.83 (0.25–2.79)
HIV Positive / reported as positive	1.12 (0.55–2.25)	1.07 (0.47–2.42)	1.97 (0.63–6.16)	2.38 (0.58–9.71)
Previous TB Treatment	1.03 (0.55–1.94)	1.16 (0.57–2.36)	1.34 (0.44–4.09)	0.73 (0.16–3.27)
Symptom screen positive	0.67 (0.40–1.13)	0.59 (0.33–1.04)	1.56 (0.66–3.69)	1.15 (0.42–3.16)
Years in healthcare	0.99 (0.97–1.01)	0.98 (0.95–1.01)	0.94 (0.89–0.99) *	0.90 (0.84–0.96)**
Home care of TB	0.73 (0.31–1.71)	0.70 (0.28–1.72)	0.99 (0.22–4.44)	0.30 (0.04–2.59)
Daily contact with TB patients	0.78 (0.39–1.5)	0.85 (0.40–1.80)	1.12 (0.25–4.99)	2.20 (0.27–17.76)

*OR* odds ratio, *CI* confidence interval

*P < 0.05; **P < 0.01; ***P < 0.001

Tables 2 and 3 presents analogous results for the analysis of discordant results between TSPOT.TB and tuberculin skin test and between QFT-GIT and TSPOT.TB, respectively.

**Table 2:** Results of multinomial regression models for discordant TSPOT.TB and tuberculin skin test at baseline

Risk Factor	Positive TST and negative TSPOT.TB test result		Negative TST and positive TSPOT.TB test result	
	OR (95% CI) *N* = 126	Adjusted	OR (95% CI) *N* = 20	Adjusted
Older age, per each additional year	0.97 (0.95–0.99) **	0.97 (0.94–0.99)*	1.00 (0.96–1.04)	1.07 (1.01–1.13)*
Male gender	0.93 (0.58–1.50)	0.72 (0.43–1.21)	1.22 (0.45–3.29)	1.46 (0.45–4.75)
BCG Vaccination scar	1.42 (0.82–2.46)	1.92 (1.02–3.63) *	1.07 (0.34–3.32)	1.04 (0.26–4.17)
HIV Positive/reported as positive	1.03 (0.50–2.11)	0.88 (0.37–2.05)	4.72 (1.64–13.59) **	4.44 (1.14–17.27)*
Previous TB Treatment	0.88 (0.46–1.68)	1.02 (0.48–2.17)	3.00 (1.09–8.28)*	1.33 (0.35–5.10)
Symptom screen positive	1.05 (0.65–1.68)	1.03 (0.60–1.75)	2.95 (1.18–7.35) *	1.94 (0.64–5.86)
Years in healthcare	0.98 (0.96–1.0)	1.00 (0.97–1.03)	CI (0.88 - 0.99)*	0.92 (0.85–0.99)*
Home-care	0.32 (0.11–0.94) *	0.32 (0.10–0.95) *	1.74 (0.48–6.30)	0.63 (0.11–3.56)
Daily contact with TB patient	0.88 (0.45–1.71)	0.94 (0.45–1.98)	2.08 (0.27–16.09)	NC

*OR* odds ratio, *CI* confidence interval

*P < 0.05; **P < 0.01; ***P < 0.001

**Table 3:** Results of multinomial regression models for discordant QFT-GIT and TSPOT.TB at baseline

	Positive QFT-GIT and negative TSPOT.TB test result		Negative QFT-GIT and positive TSPOT.TB test result	
Risk Factor	OR (95% CI) *N* = 47	Adjusted	OR (95% CI) *N* = 29	Adjusted
Older age, per each additional year	95% CI ; 0.94 - 0.99) *	0.99 (0.95–1.03)	0.97 (0.92–1.01)	1.03 (0.99–1.08)
Male gender	0.98 (0.50–1.97)	0.98 (0.47–2.05)	1.09 (0.47–2.55)	1.54 (0.57–4.13)
BCG Vaccination scar	0.66 (0.33–1.33)	0.88 (0.37–2.01)	0.54 (0.22–1.15)	0.48 (0.19–1.23)
HIV Positive/reported as positive	1.18 (0.44–3.18)	1.66 (0.53–5.24)	5.11 (2.11–12.33) ***	5.72 (1.95–16.78)**
Previous TB Treatment	0.29 (0.07–1.22)	0.24 (0.05–1.17)	1.34 (0.49–3.67)	0.74 (0.23–2.36)
Symptom screen positive	1.35 (0.70–2.59)	1.48 (0.71–3.08)	1.09 (0.47–2.55)	0.78 (0.29–2.11)
Years in healthcare	0.97 (0.93–1.00)	0.97 (0.93–1.02)	0.97 (0.93–1.01)	0.95 (0.90–1.01)
Home-care	0.26 (0.03–1.94)	0.24 (0.03–1.85)	3.10 (1.17–8.21) *	2.42 (0.77–7.56)
Daily contact with TB patient	0.59 (0.24–1.40)	0.71 (0.27–1.87)	0.64 (0.21–1.94)	1.04 (0.27–4.00)

*OR* odds ratio, *CI* confidence interval

*P < 0.05; **P < 0.01; ***P < 0.001

#### BCG

A TST + ve/IGRA-ve discordant test response was more likely in those with a BCG vaccination scar and significantly so for TST + ve/TSPOT.TB-ve. BCG scar positive Individuals were less likely to have QFT-ve/TSPOT.TB + ve discordance.

#### Length of service

HCWs with more years in healthcare employment were less likely to have a discordant test (TST-ve/IGRA+ve) outcome; in other words, TST/IGRA discordance declined with duration of employment.

#### HIV status

HIV infected individuals were more likely to test TSPOT.TB + ve/ TST-ve and TSPOT.B + ve /QFT-GIT-ve, relative to HIV negative HCWs.

#### Home-based care

HCWs engaged in home care were less likely to have a discordant test (TST + ve/ TSPOT.TB-ve) response.

## Discussion

This study evaluated factors associated with discordant test responses for presumed latent TB infection in healthcare workers in a high TB and HIV prevalence setting. It found the following factors significantly associated with an increased odds of discordance: HIV infection, long exposure in healthcare environments and BCG vaccination. The finding of a markedly increased odds of a discordant test response in HIV-infected HCWs (OR = 4.44) for TSPOT.TB + ve/TST-ve and (OR = 5.72) for TSPOT.TB + ve/QFT- GIT-ve suggests that TSPOT.TB test performance is relatively unimpaired by HIV infection in this population and is therefore a potentially more sensitive assay than TST for detecting LTBI in HIV-infected HCWs [16, 17]. A meta- analysis by Cattamanchi et al. did not show IGRAs as a class to be consistently more sensitive than TST in HIV-infected individuals but did conclude that the TSPOT.TB assay exhibited higher sensitivity and was less affected by immunosuppression, than either TST or QFT-GIT [18]. A subsequent review by Overton et al. similarly concluded that the rate of reactivity of both IGRAs and TST was affected by the level of HIV-associated CD4 T-cell deficiency and that all assays appeared to be affected by the degree of CD4 T-cell depletion, with the possible exception of the TSPOT.TB assay [19, 20]. It is noted however that studies included in the systematic reviews were heterogeneous, few performed head to head comparisons between TST and IGRAs, and there were few studies specifically evaluating the performance of TSPOT. TB. As suggested by the epidemiological finding in this study, the performance of TST and QFT- GIT appears to be more susceptible to CD4 T cell depletion, manifesting as anergic TST responses and low-mitogen QFT-GIT responses with CD4 count acting as an effect modifier. Overton et al. further conclude that the likelihood of a positive reaction in the TST or ELISA-based IGRA at low CD4 levels is negligible.

Alternative explanations for the IGRA-TST and IGRA-IGRA discordance may include the test format (ELISPOT is a cell-based readout and therefore inherently more sensitive than ELISA), the use of different test-specific antigen formulations that may differentially turn on regulatory (inhibitory) pathways, host factors, strain type, or likely a combination of these factors [21, 22]. Emerging research suggests that different *M. tb* strains differentially regulate host IFN-gamma responses in HIV negative individuals and that these differences may be a result of genetic differences in the ESX-1 region. This could in part explain some of the differences seen in test responses to different IGRA assays [23].

However in the absence of diagnostic certainty and a gold standard for LTBI, it remains difficult to conclusively assume increased sensitivity of IGRAs compared to TST.

The significantly decreased odds of a TSPOT.TB-ve/ TST + ve discordance in home- carers (after controlling for HIV) is of relevance. Community health workers comprise a large and growing population of health workers and form the backbone of the TB control programmes in their role as treatment and adherence supporters through home visits, often in impoverished settings including informal housing settlements. Drawn from the same communities as their clients, their risk for both TB and HIV has been shown to be greater than that of the general population [24].

The decrease in TST-ve / IGRA+ve discordance with duration of employment is not unexpected as increased risk of LTBI has been associated with increased length of employment in several studies for both IGRAs and TST [25, 26]. The underlying mechanism of this duration effect may be one in which IGRA is more likely than TST to detect recent infection (involving CD4 T-cells that have encountered antigens recently in vivo), whereas TST detects cumulative exposure to M. tuberculosis over time (involving CD4 T- cells of the memory phenotype) [27, 28]. In this study BCG vaccination (as assessed by presence of a vaccination scar) was positively associated only with a TST + ve/TSPOT.TB- ve outcome and not with TST + ve/QFT-ve discordance, suggesting an effect on TST test response. This is similar to a large study in Chinese healthcare workers, which showed higher rates of LTBI as measured by TST in those with vaccination scars [29]. However, not all studies have found an association between discordance and BCG vaccination given at birth only, with at least two reviews reporting that the influence of BCG is relatively limited in the adult population 10 years or more after BCG vaccination [22, 30].

There are several limitations to this study. Voluntary participation and limited participation by clinical staff due to high workload may have resulted in selection bias. The high rate of indeterminate results for TSPOT.TB also resulted in a smaller sample being available for analysis of discordance for this assay. The lack of data on extent of immunosuppression as reflected by CD4 is another limitation. This information could have enhanced our understanding of the spectrum of IGRA test response in those who are HIV infected. The use of standardized cut-offs to denote test positivity is another factor that may influence some of the associations shown. The widespread use of IGRAs in serial testing programmes have highlighted the variability of these assays and the need for a more cautious approach to interpretation of test positivity for values close to the cut-off [31]. An exploration of different TST and IGRA cut points may inform a more nuanced understanding of factors associated with discordance. Lastly the lack of a gold standard to confirm LTBI diagnosis makes it difficult to compare test performance and arrive at conclusive answers in this regard.

## Conclusion

In this population BCG vaccination is associated with discordance suggesting an enduring effect for BCG given at birth and likely impact on TST test specificity. The markedly discordant test response in HIV infected HCWs found in this study is compatible with potentially greater sensitivity or reactivity of TSPOT.TB in immunocompromised individuals. Whilst it is known that both IGRAs and TST are only modestly predictive of subsequent TB risk, LTBI diagnosis and treatment remains one of the interventions recommended by the World Health Organization (WHO) to end the TB epidemic worldwide [32, 33]. In high-incidence countries it is imperative that such screening selectively and accurately targets those at sufficiently high risk of progressing to disease.

A potentially increased test sensitivity of TSPOT.TB in a setting of a high prevalence of HIV would allow for more targeted IPT for those at greatest risk of progression to disease. This may enhance its potential utility in a TB and HIV endemic setting where at least 10% of HCWs are HIV-infected and face a six-fold higher risk of contracting TB, as has been shown to be the case among HCWs working in South African hospitals [34].

## Abbreviations

LCA: Latent class analysis
LTBI: Latent tuberculosis infection
QFT-GIT: QuantiFERON-gold-in-tube
TB: Tuberculosis
TST: Tuberculin skin test

## References

[1] Dheda K, Barry CE, Maartens G (2016). Tuberculosis. Lancet.

[2] Latent tuberculosis infection (2018). updated and consolidated guidelines for programmatic management.

[3] Denkinger CM, Dheda K, Pai M. Guidelines on interferon-c release assays for tuberculosis infection: concordance, discordance or confusion? Clinical Microbiology and Infection ª2011 European Society of Clinical Microbiology and Infectious Diseases, CMI, 17, 806–814. 10.1111/j.1469-0691.2011.03555.x 21682801

[4] Grobler L, Mehtar S, Dheda K, Adams S, Babatunde S, van der Walt M, et al. The epidemiology of tuberculosis in health care workers in South Africa: a systematic review. BMC Health Serv Res. 2016;16(1) 416–016-1601. 10.1186/s12913-016-1601-5 PMC4992336 27544429

[5] South Africa, Department of Health (2014). National tuberculosis management guidelines.

[6] Sommerland N, Wouters E, Masquillier C, Engelbrecht M, Kigozi G, Uebel K, Janse van Rensburg A, Rau A (2017). Stigma as a barrier to the use of occupational health units for tuberculosis services in South Africa. Int J Tuberc Lung Dis.

[7] Menzies D, Pai M, Comstock G (2007). Meta-analysis: new tests for the diagnosis of latent tuberculosis infection: areas of uncertainty and recommendations for research. Ann Intern Med.

[8] Pai M, Gokhale K, Joshi R, Dogra S, Kalantri S, Mendiratta DK (2005). *Mycobacterium tuberculosis* infection in health care workers in rural India: comparison of a whole-blood interferon gamma assay with tuberculin skin testing. JAMA.

[9] Pai M, Kalantri S, Menzies D (2006). Discordance between tuberculin skin test and interferon- gamma assays. Int J Tuberc Lung Dis.

[10] Pai M (2005). Alternatives to the tuberculin skin test: interferon-gamma assays in the diagnosis of *Mycobacterium tuberculosis* infection. Indian J Med Microbiol.

[11] National Institute for Health and Clinical Excellence (NICE). Tuberculosis: clinical diagnosis and management of tuberculosis, and measures for its prevention and control. 2006; NICE Clinical Guidelines, No. 33. 22720337

[12] Garone D, Hilderbrand K, Boulle A, Coetzee D, Goemaere E, Van Cutsem G, Besada D (2011). Khayelitsha 2001–2011: 10 years of primary care HIV and TB programmes. Southern African J HIV Med.

[13] Adams S, Ehrlich R, Baatjies R, van Zyl-Smit RN, Said-Hartley Q, Dawson R, Dheda K (2015). Incidence of occupational latent tuberculosis infections in south African healthcare workers. Eur Respir J.

[14] van Zyl-Smit RN, Pai M, Peprah K, Meldau R, Kieck J, Juritz J (2009). Within-subject variability and boosting of T-cell interferon-gamma responses after tuberculin skin testing. Am J Respir Crit Care Med.

[15] van Zyl-Smit RN, Zwerling A, Dheda K, Pai M (2009). Within-subject variability of interferon-g assay results for tuberculosis and boosting effect of tuberculin skin testing: a systematic review. PLoS One.

[16] Rangaka MX, Diwakar L, Seldon R, van Cutsem G, Meintjes GA, Morroni C (2007). Clinical, immunological, and epidemiological importance of antituberculosis T cell responses in HIV-infected Africans. Clin Infect Dis.

[17] Rangaka MX, Wilkinson KA, Seldon R, Van Cutsem G, Meintjes GA, Morroni C (2007). Effect of HIV-1 infection on T-cell-based and skin test detection of tuberculosis infection. Am J Respir Crit Care Med.

[18] Cattamanchi A, Smith R, Steingart KR, Metcalfe JZ, Date A, Coleman C (2011). Interferon-gamma release assays for the diagnosis of latent tuberculosis infection in HIV- infected individuals: a systematic review and meta-analysis. J Acquir Immune Defic Syndr.

[19] Overton K, Varma R, Post JJ (2018). Comparison of interferon-γ release assays and the tuberculin skin test for diagnosis of tuberculosis in human immunodeficiency virus: a systematic review. Tuberc Respir Dis.

[20] Ayubi E, Doosti-Irani A, Moghaddam AS, Sani M, Nazarzadeh M, Mostafavi E (2016). The clinical usefulness of tuberculin skin test versus interferon-gamma release assays for diagnosis of latent tuberculosis in HIV patients: a Meta-analysis. PLoS One.

[21] Davids M, Pooran AS, Meldau R, Thompson F, Gina P, Dheda K. A Human Lung-Orientated Approach to Correlates of Risk in Tuberculosis. B63. Latent tuberculosis infection and epidemiology of tuberculosis. 2017 A3994-A3994.

[22] Pai M, Denkinger CM, Kik SV, Rangaka MX, Zwerling A, Oxlade O (2014). Gamma interferon release assays for detection of mycobacterium tuberculosis infection. Clin Microbiol Rev.

[23] Tomasicchio M, Limberis J, van der Merwe R, Warren R, Meldau R, Theron G, Nicol M, Jacobson R, Helden P, Dheda K. *Mycobacterium tuberculosis* strains from endemic settings are genotypically different in the ESX-1 region likely explaining the differential modulation of IFN-g-specific host interferon-gamma responses. (in preparation).

[24] Kranzer K, Bekker L, van Schaik N, Thebus L, Dawson M, Caldwell J, Hausler H, Grant R (2010). Community health care workers in South Africa are at increased risk for tuberculosis. S Afr Med J.

[25] Rafiza S, Rampal KG, Tahir A (2011). Prevalence and risk factors of latent tuberculosis infection among health care workers in Malaysia. BMC Infect Dis.

[26] van Rie A, Mc Carthy K, Scott L, Dow A, Venter WDF, Stevens WS (2013). Prevalence , risk factors and risk perception of tuberculosis infection among medical students and healthcare workers in Johannesburg, South Africa. S Afr Med J.

[27] Pai M, Riley LW, Colford Jr JM. Interferon- assays in the immunodiagnosis of tuberculosis: a systematic review .2004. Lancet Infect Dis ; 4: 761–776. 10.1016/S1473-3099(04)01206-X 15567126

[28] Hesseling AC, Mandalakas AM, Kirchner HL, Chegou NN, Marais BJ, Stanley K, Zhu X, Black G, Beyers N, Walz G. Highly discordant T cell responses in individuals with recent exposure to household tuberculosis Thorax 2009; 64:840–846. doi:10.1136/thx.2007.085340. 10.1136/thx.2007.085340 18682523

[29] He GX, van den Hof S, van der Werf MJ, Wang GJ, Ma SW, Zhao DY (2010). Infection control and the burden of tuberculosis infection and disease in health care workers in China: a cross-sectional study. BMC Infect Dis.

[30] Wang L, Turner MO, Elwood RK, Schulzer M, FitzGerald JM (2002). A meta-analysis of the effect of Bacille Calmette Guérin vaccination on tuberculin skin test measurements. Thorax.

[31] Tagmouti S, Slater M, Benedetti AV, Kik S, Banaei N, Cattamanchi A, Metcalfe J, Dowdy D, van Zyl Smit R, Dendukuri N, Pai M, and Denkinger C. Reproducibility of Interferon Gamma (IFN-g) Release Assays A Systematic Review Ann Am Thorac Soc Vol 11, No 8, pp 1267–1276, 2014. 10.1513/AnnalsATS.201405-188OC PMC5469356 25188809

[32] Pai M, Behr M. 2016. Latent mycobacterium tuberculosis infection and interferon- gamma release assays. Microbiol Spectrum 4(5): TBTB2–0023-2016. 10.1128/microbiolspec.TBTB2-0023 -2016.) 10.1128/microbiolspec.TBTB2-0023-2016 27763261

[33] World Health Organization. 2014. The end TB strategy. Global strategy and targets for tuberculosis prevention, care and control after 2015. https://www.who.int/tb/post2015_TBstrategy.pdf.

[34] Tudor C, van der Walt ML, Margot B (2016). Occupational Risk Factors for Tuberculosis Among HCWs •. Clin Infect Dis.

